# Exploring marking methods for the predatory hoverfly *Sphaerophoria rueppellii* (Diptera: Syrphidae)

**DOI:** 10.1111/1744-7917.70020

**Published:** 2025-03-16

**Authors:** Michele Violi, Elena Costi, Elena Monari, Daniele Sommaggio, Lara Maistrello

**Affiliations:** ^1^ Department of Life Sciences University of Modena and Reggio Emilia Via G. Amendola 2 Reggio Emilia 42122 Italy; ^2^ National Biodiversity Future Center (NBFC) Piazza Marina 61 Palermo 90133 Italy

**Keywords:** biocontrol, fluorescein, fluorescent dusts, hoverflies, insect tagging, rubidium

## Abstract

Hoverflies are essential to ecosystems, with adults serving as important pollinators and larvae preying on plant‐feeding insects or recycling nutrients. Species like *Sphaerophoria rueppellii* are used in biocontrol programs to target aphid pests. To enhance these programs, markers can be used in a mark‐release‐recapture (MRR) method to track hoverfly feeding and oviposition sites. Effective markers must be persistent and not harm the hoverflies’ vital functions. This study evaluated three marking methods for *S. rueppellii*: rubidium (RbCl), fluorescein, and fluorescent dust. Laboratory experiments assessed the effects of these markers on female hoverfly fecundity, mating behavior and marking persistence. Results showed no significant differences in egg‐laying or survival time between marked and unmarked females. Rubidium and fluorescein did not affect mating behavior, but dust‐treated females mated significantly less than untreated females. In terms of marking persistence, rubidium and fluorescent dusts remained detectable throughout the hoverflies’ adult lifespan, while fluorescein markings faded within 24 h. Fluorescent dusts were easy‐to‐use, durable, and cost‐effective, but careful application and further study are needed to avoid potential effects on insect activity and mating ability. Fluorescein showed no adverse effects on insect biology, was economical and quick to apply, but had short persistence, making it unsuitable for long‐term field studies. Rubidium was harmless to insects and detectable for long periods, but its detection required financial investment, time, and specialized equipment. This research provides valuable insights into the potential of hoverflies as biocontrol agents and offers new tools for their effective management in agricultural settings.

## Graphical abstract

As important pollinators and predators of aphids, hoverflies play an important role in ecosystems. This study focuses on identifying the best marking technique for the model species *Sphaerophoria rueppellii* that can be used to track hoverfly feeding and oviposition sites, evaluating three methods: rubidium (RbCl), fluorescein, and fluorescent dusts. Results showed no significant differences in oviposition or survival time between marked and unmarked females. In terms of marking persistence, rubidium and fluorescent dusts remained detectable throughout the adult life of the hoverflies, while fluorescein markings faded within 24 h. While rubidium and fluorescein did not interfere with mating behavior, dust‐treated females mated significantly less than untreated females.

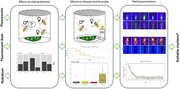

## Introduction

Approximately one third of hoverfly species (Diptera: Syrphidae) are predators of aphids during the larval stage and thus contribute significantly to the biological control of important pests of many crops, both in the open field and in greenhouses (Pekas *et al.*, 2020; Rodríguez‐Gasol *et al.*, 2020). Some hoverflies, such as *Sphaerophoria rueppellii* (Wiedemann, 1820) and *Eupeodes corollae* (Fabricius, 1794) in Europe and *Eupeodes americanus* (Wiedemann, 1830) in North America, are purposely reared in biofactories for specific augmentative biocontrol programs (Pekas *et al.*, 2020; Bellefeuille *et al.*, 2021; Li *et al.*, 2023; Burgio *et al.*, 2024; Fauteux *et al.*, 2024; Gonzalez *et al.*, 2024; Ouattara *et al.*, 2024).

The main limitation of the use of hoverflies in agriculture is that the commercially available stage is the pupa, whereas the larvae are the real biocontrol agent, thus the delay to take action often require preventive releases. Also, the adults feed on pollen and nectar (Rodríguez‐Gasol *et al.*, 2020; Burgio *et al.*, 2024), not always available from crops or in extremely simplified agro‐ecosystems. Since the hoverflies are excellent flyers, and numerous species show migratory behavior (Odermatt *et al.*, 2017), it is likely that the adults disperse by moving away from the release site, especially in the absence of optimal conditions such as food for the adults and presence of aphid infestation where the females can lay eggs (Rodríguez‐Gasol *et al.*, 2020; Burgio *et al.*, 2024).

Some measures can be adopted to favor the persistence of hoverflies in the release area in order to improve the biological control of aphid populations. The introduction of flower strips in agroecosystems is one potential solution, as they provide a food source for adults (Pineda & Marcos‐Garcia, 2008b; Haenke *et al.*, 2009; Jönsson *et al.*, 2015; Tschumi *et al.*, 2016). Another option is the use of banker plants, that is, the use of secondary plants that support the reproduction or persistence of a biocontrol agent by providing alternative prey that is not harmful to the crop; the use of banker plants has shown promise (Bellefeuille *et al.*, 2021; Fauteux *et al.*, 2024; Gonzalet *et al.*, 2024).

Understanding the movement patterns of released adults is crucial for assessing the effectiveness of biological control programs, particularly with respect to their ability to remain within the target area. Although larvae are important for pest control, they do not face the same risk of dispersal, making adults the critical stage for this study. The Mark‐Release‐Recapture (MRR) technique could be a useful tool for exploring this area of research. A variety of insect marking techniques have been used in MRR methods to study a range of ecological relationships, including population dynamics, dispersal, territoriality, feeding habits and trophic interactions (Grimm *et al.*, 2014). An ideal marker should fulfill its role without disrupting the insect's normal biological functions, and be environmentally friendly, affordable, persistent and easy to apply and detect. In insects, MRR techniques have been used mainly in a few very charismatic groups such as butterflies (e.g., Schtickzelle *et al.*, 2002), beetles (e.g., Roslin, 2000), bees (e.g., Briggs *et al.*, 2022) and dragonflies (e.g., Khelifa *et al.*, 2021). The most commonly used markers in insects are dyes, dusts, paint tags, mutilations, trace elements and proteins (Hagler & Jackson, 2001).

Mark and recapture studies have also been conducted on hoverflies, primarily to investigate migrations (Aubert & Goeldlin de Tiefenau, 1981), population size and longevity (Nielsen, 1969; Conn, 1976; Ball & Morris, 2004), male hill topping behavior (Alcock, 2011), dispersal and habitat utilization in natural environments (Holloway & McCaffery, 1990; Wolton *et al.*, 2012; Rotheray *et al.*, 2014), movements in agroecosystems in relation to floral resources and barriers (Lövei *et al.*, 1998; MacLeod, 1999; Wratten *et al.*, 2003), and retention strategies for hoverflies released in greenhouses (Pineda & Marcos‐Garcia, 2008c). Paint tagging of adults is the most common marking methods adopted in these studies, specifically acrylic paint (MacLeod, 1999; Wolton *et al.*, 2012), enamel paint (Holloway & McCaffery, 1990; Ball & Morris, 2004; Rotheray *et al.*, 2014), water‐based paint (Pineda & Marcos‐Garcia, 2008c), cellulose paint (Conn, 1976), sprayed nitrofluorescent varnish (Aubert & Goeldlin de Tiefenau, 1981), paint pens (Alcock, 2011) and dry pigment mixed with glue (Nielsen, 1969). Pollen of strategically planted species has also been used as a marker (Lövei *et al.*, 1998; Wratten *et al.*, 2003). In laboratory experiments, Dinkel & Lunau (2001) used small numbered tags to study how hoverflies use floral guides to locate food sources, and Barlow (1961) employed wing clipping to study survival and oviposition rates.

The aim of this study was to test further marking methods that could be employed to understand the movements of adult predatory hoverflies, useful for example to evaluate the effectiveness of using flower strips or banker plants. The trace element rubidium, fluorescein dye and fluorescent dusts were selected for this investigation, because they have been widely used to track other insects with successful results. Rubidium has been used either to mark insects directly via enriched food in the laboratory (Graham & Wolfenbarger, 1977; Van Steenwyk *et al.*, 1978; Knight *et al.*, 1989; Jost & Pitre, 2002; Maciel‐de‐Freitas *et al.*, 2004) or sprayed in the field on crops, host plants, food resources, or shelters (Corbett *et al.*, 1996; Prasifka *et al.*, 2001; Klick *et al.*, 2015; MacKinnon *et al.*, 2016; Madeira & Pons, 2016). Since rubidium occurs naturally in the environment at low concentrations, background levels of the species or population being studied must be assessed to ensure marking effectiveness (Hagler & Jackson, 2001).

Fluorescein is a water‐soluble dye that is detectable at a dilution of 0.02 ppm under UV light and belongs to the fluorescent dye family of markers. It has already been successfully used to mark insects internally through food for Diptera such as adult mosquitoes (Sarkar *et al.*, 2017) and Calliphoridae (Coppedge *et al.*, 1979). Bribosia *et al.* (2005) effectively labeled *Episyrphus balteatus* (De Geer) (Diptera: Syrphidae) eggs by feeding females with a solution of Rhodamine B, a similar fluorescent dye.

Fluorescent dusts (also referred to as “powders”) are among the most commonly used methods for externally marking insects (Nakata, 2008; Dickens & Brant, 2014; Clymans *et al.*, 2020; Kirkpatrick *et al.*, 2020; Nixon *et al.*, 2021). Dusts are affordable, available in a range of colors, detectable under UV light and can be applied directly to insects or mixed with water and sprayed (Hagler & Jackson, 2001). However, excessive application can reduce mobility and interfere with sensory or respiratory organs, particularly in small and lightweight species, for example small dipterans (Messing *et al.*, 1993; Culbert *et al.*, 2020).

The model species selected for this study is *S. rueppellii*, as it is one of the most common predatory hoverflies found in Mediterranean greenhouses and it is also commercially available as a biocontrol agent for the management of aphid populations (Pineda & Marcos‐García, 2008a, 2008b; Amorós‐Jiménez *et al.*, 2012; Burgio *et al.*, 2024). The larvae of this species are considered generalist predators, feeding on a wide range of aphid species as well as some species of thrips, spider mites, and whiteflies (Rotheray & Gilbert, 1989; Rojo *et al.*, 2003). In recent years, its biology has been extensively studied (Amorós‐Jiménez *et al.*, 2012; Amorós‐Jiménez & Marcos‐García, 2020; Orengo‐Green *et al.*, 2022; Leman *et al.*, 2023). In this study, marker techniques were tested exclusively on females, as their retention at the release site is of primary importance to ensure oviposition on the crop and could be the focus of potential future field applications.

## Materials and methods

### Insects rearing

Adult *S. rueppellii* were obtained from pupae supplied by Koppert (Spain). Upon arrival, the pupae were maintained at room temperature (25 ± 1 °C) until the emergence of the adults. If experiments required unmated adults, individual pupae were kept in 50 mL Falcon tubes with gauze and a drop of honey until emergence. Otherwise, pupae were kept together on a Petri dish in a 30 cm × 30 cm × 30 cm BugDorm cage and provided with a solution of 2 : 10 w/w sugar and water in a 50 mL Falcon tube with a dental cotton on the lid until the marking procedure. Cotton aphids, *Aphis gossypii* (Glover, 1877) (Hemiptera: Aphididae), were collected in the spring of 2024 on zucchini (*Cucurbita pepo* L.) seedlings at a local garden center (44°38′39.6″N, 10°40′08.4″E, Reggio Emilia, Italy) and reared on potted plants of the same species in a climatic chamber at 26 ± 1 °C, with a 16 : 8 (L : D) photoperiod and 70% relative humidity (RH).

### Markers application

Preliminary trials were conducted to determine the lowest effective concentration of RbCl and fluorescein and a suitable method of application for the dusts, that is, that would show sufficient persistence and presumably, cause the least collateral effects on the life parameters of *S. rueppellii*.

Fluorescein sodium salt (Carlo Erba Reagents, Val de Reuil Cedex, France) and rubidium chloride (RbCl) (ITW Reagents, Monza, Italy) were administered via the food supply, added to a solution of 2 : 10 w/w of sugar and water provided in a 50 mL falcon tube with a dental cotton inserted in a hole drilled in the lid. One and two days old insects were maintained on the enriched diets in a 30 cm × 30 cm × 30 cm BugDorm cage for a period of 48 h prior to the start of the experiments. For rubidium, a 0.01 mol/L RbCl solution (= 1.2 g RbCl per L of food solution = 1211 ppm of RbCl = 856 ppm of Rb) was selected. For fluorescein, a concentration of 0.01% of the food solution was selected, which was also adopted in the studies by Coppedge *et al.* (1979) and Sarkar *et al.* (2017). Successfully marked hoverflies showed fluorescence in the ventral part of the abdomen. In contrast to the findings of Coppedge *et al.* (1979), higher concentrations of fluorescein did not appear to improve the persistence or visibility of the marking in *S. rueppellii* in the preliminary trials. Attempts to externally mark adults with fluorescein, for instance using a sprayer as in Argauer & Cantelo (1972), yielded unsatisfactory results in terms of fluorescence detectability.

Orange fluorescent dust (Ebanku^®^, Guangdong, China) was applied to the thorax of adult hoverflies using a small brush. The hoverflies were immobilized using a handmade device, similar to that used for marking queen bees, consisting of a modified 9 cm Petri dish with a 7 cm diameter hole in the valve of the container and covered with mesh. The lid valve was inverted to serve as a base. The hoverflies were then carefully caught and immobilized between the mesh and the lid and dusted with the brush through the mesh. This marking procedure was performed 24 h prior to the beginning of experiments. A commonly used marking method in the literature was to add a variable amount of dust to a container with the insects, which was then shaken. This method, adopted for example with psyllids (Nakata, 2008), mosquitoes (Dickens & Brant, 2014; Culbert *et al.*, 2020) and fruit flies (Clymans *et al.*, 2020), was discarded after high mortality rates were observed in preliminary trials.

### Effects on mating behavior

The effects on mate choice were assessed in cylindrical transparent plastic containers, 8 cm high and 10 cm in diameter, surrounded by fine mesh at the top. Each arena contained one unmated male exposed to two unmated females: one marked and one unmarked, which served as a control. Several replicates were performed for each marking method (fluorescein *n* = 28, dusts *n* = 29, rubidium *n* = 31). A zucchini seedling infested with *A. gossypii* was added to each arena to provide support and to stimulate mating. Arenas were kept in a climate‐controlled chamber (26 ± 1 °C, 70% RH) for a maximum of 7 h, from morning to afternoon, and monitored by scan sampling at 15‐min intervals to ascertain whether mating events occurred.

To determine whether the male hoverfly preferred the treated or untreated female, a UV light torch (395 nm, COSOOS, MA, USA) was used to distinguish between the fluorescein‐ and dust‐treated females and the untreated ones. For rubidium (Rb) assays, both females were analyzed for Rb content, to incontrovertibly determine whether males chose treated or untreated females, thus avoiding errors due to the possibility that RbCl‐treated diet‐fed females did not actually consume the diet or that untreated females were subjected to potential RbCl contamination. Hoverflies sampled for Rb content were killed and stored at −18 °C until the analysis. Before digestion, the insects were weighed using an Entris 224‐1S analytical balance (Sartorius, Göttingen, Germany), placed individually in 5 mL tubes, and dissolved in 0.5 mL of concentrated supra‐pure HNO_3_. The samples were then heated in a Falc Thermoblock TD 200 P2 at 65 °C for 2 h. After cooling, ultrapure water was added to obtain a 5 mL of final solution. A further 1 : 50 dilution in ultrapure water of the samples was made prior to the analysis for Rb content using an iCAP TQ IPC‐MS spectrometer (Thermo Fisher Scientific, MA, USA) performed at the Centro Interdipartimentale Grandi Strumenti (Modena, Italy) of the University of Modena and Reggio Emilia. The concentration of Rb in the samples was then expressed in terms of insect weight to obtain a concentration in ppm per insect.

### Effects on female survival and fecundity

To assess the effect of marking treatment on the life span and fecundity of *S. rueppellii* females, ten replicates were conducted for each marking method and for the untreated control (rubidium, *n* = 10; fluorescein, *n* = 10; dust, *n* = 10; control, *n* = 10). Following the 48‐h marking period (during which the untreated and dust‐treated females and all the males were fed on a solution of sugar and water only at 2 : 10 w/w), one unmated female and one unmated male were placed in each experimental unit, which consisted of a 10 cm × 10 cm × 15 cm transparent plastic container, closed at the top with a mesh. A 9 cm Petri dish with a *C. pepo* leaf on technical agar infested with *A. gossypii* was placed in each container. The leaves on the Petri dishes were prepared and infested with aphids 2 d prior to administration, allowing the aphids to multiply in a climatic chamber (26 ± 1 °C, 16 L : 8 D, 70% RH). Only leaves containing at least 30 aphids were administered to the experimental units. Each replicate was also provided with a 5 mL tube containing a dental cotton covered with pollen (Il Pungiglione s.c.s., Tuscany, Italy), placed in a hole drilled in the lid and filled with a 2 : 10 w/w honey and water solution used as food. The tube of food solution and the Petri dish with infested leaves were replaced three times a week. At the same time, the number of eggs laid and the number of dead females were counted. The longevity of the females was calculated as the number of days between the beginning of the experiment, after the marking period, and their death. Dead males were removed. Matings were not recorded, but males found dead at the first check (48 h after setting up the experiment) were replaced with another unmated male of the same age to ensure that individuals had sufficient time to copulate. For example, *E. corollae* was found to copulate 2 d after hatching (Zheng *et al.*, 2019) or on the day of hatching (Benestad, 1970). *S. rueppellii* males are sexually mature by the second day after hatching when kept at 25 °C (Orengo‐Green *et al.*, 2022). It should be noted that the flies were at least 2 d old at the start of the experiment. Observations continued until the female died.

### Marking persistence

Following the marking procedure, 30 RbCl‐treated females (*n* = 30), 30 untreated females fed sugar and water only (acting as an untreated control) (*n* = 30), 20 fluorescein‐treated females (*n* = 20) and 30 dust‐marked females (*n* = 30) were maintained in 30 cm × 30 cm × 30 cm BugDorm cages with one 50 mL falcon tube per 10 individuals with 2 : 10 w/w sugar and water and one Petri dish with 5 g of granular pollen (Il Pungiglione s.c.s., Tuscany, Italy).

Three live individuals (*n* = 3) from the RbCl‐treated and untreated groups were collected three times per week and analyzed for Rb content as described above. Dead individuals were discarded. Individuals were collected until no survivors were left.

For fluorescein‐treated and dust‐treated females, 5 live individuals (*n* = 5) were randomly collected and kept in a Petri dish at 4 °C for 2 min to reduce movement. The hoverflies were then observed under an Optika SZX‐T stereomicroscope with a UV light illuminator. Fluorescein‐treated individuals were recorded as either marked or unmarked, depending on whether they could be distinguished from unmarked individuals. Each dust‐marked individual was photographed using a Bresser Mikrokular camera attached to the ocular of the stereomicroscope. The images were then analyzed using the ImageJ 1.54g image processing software to quantify the percentage of the body area covered by dust. Collection of fluorescein‐treated females was continued until none of the remaining individuals exhibited fluorescence, while collection of dust‐treated females was continued until all individuals died.

### Data analysis

Deviations from normality were identified by histograms and Q‐Q plots, while nonconstant variance was detected by scatterplots of residuals versus fitted values.

A Fisher's Exact test was performed to assess whether male hoverflies showed a preference for treated or untreated females. This analysis allowed us to test the null hypothesis that males showed no preference between treated and untreated females. The test was conducted using a contingency table, comparing the observed frequencies of male approaches to each group with the frequencies expected under the assumption of no preference.

A nonparametric Kruskal‐Wallis test was used to compare the number of eggs laid by hoverflies in the different treatments. A Kaplan–Meier survival curve and associated risk table were used to estimate and visualize the probability of survival over time for treated and untreated hoverflies. The survival curves of hoverflies exposed to different treatments were compared using a log‐rank test. The effect of species on survival over time was assessed using the Cox regression model.

To evaluate the persistence of the marking powder and rubidium over time, persistence trends were assessed qualitatively by plotting the percentage of powder retained on the insects and the rubidium concentration (ppm) over a 15‐d period.

All analyses were performed with R software 4.4.1. A significance level of *α* = 0.05 was used for each test.

## Results

### Effects on mating behavior

Only a percentage of males mated on the day of observation (fluorescein = 67.9%, *n* = 28; rubidium = 45.2%, *n* = 31; fluorescent dust = 44.4%, *n* = 29). When choices were made, treated females mated in 57.89%, 42.86%, and 21.43% of cases over untreated females in fluorescein, rubidium, and dust groups.

The results of Fisher's Exact Test, detailed in Table 1, indicate that males did not differ significantly in their choice between treated and untreated females in the case of rubidium and fluorescein treatments, but fluorescent dust‐treated females mated significantly less often than untreated females (*P* = 0.008).

**Table 1:** Percentage of male choices (untreated vs. treated), number of observations (num), and *P*‐values from *χ* ^2^ test for each treatment (fluorescein, rubidium, and fluorescent dust)

	Untreated	Treated	num	*P*‐value
Fluorescein	42.11	57.89	19	0.516
Rubidium	57.14	42.86	14	0.706
Fluorescent dust	78.57	21.43	14	0.008**

The *P*‐values indicate the significance of the differences between untreated and treated groups for each predictor. **P < 0.01.

### Effects on female survival and fecundity

RbCl‐treated females laid more eggs than other groups, with a median of 63 eggs/female (mean = 110.2; max = 344; *n* = 10), followed by the control group (median = 18; mean = 41.4; max = 166; *n* = 10), fluorescein‐treated females (median = 4.5; mean = 51.5; max = 327; *n* = 10), and by fluorescent dust‐treated females (median = 2; mean = 57.0; max = 414; *n* = 10). Some females died prematurely during the first few days without laying eggs, in numbers of 3, 5, 3, and 3 for rubidium, dusts, fluorescein and control groups respectively. No significant differences were detected between groups when comparing the number of eggs laid per female with Kruskal‐Wallis test (*χ* ^2^ = 1.904, df = 3, *P* = 0.593). Descriptive statistics of the median [IQR] number of eggs laid by females exposed to different treatments are reported in Table 2.

**Table 2:** Median [IQR] number of eggs laid by treated (fluorescein, fluorescent dust, rubidium) and untreated (control) hoverfly females

Treatment	Median [IQR]	Num.
Control	18 [0.25, 30.5]	10
Fluorescein	4.5 [0.25, 36.5]	10
Fluorescent dust	2 [0, 24]	10
Rubidium	63 [2.5, 196.5]	10

Num. = the number of replicates for each treatment.

Females survived an average of 6.5 d in rubidium, 6.8 d in fluorescent dust, 6.0 d in fluorescein, and 5.4 d in the control treatment. The longest surviving female lived up to 12 d in rubidium and control treatments, 14 d in fluorescein treatment and 16 d in dust treatment. The results of the Cox Proportional Hazards Model are presented in Table 3. For the fluorescein treatment, the hazard ratio is approximately 1.385, indicating that the mortality risk for this cohort is approximately 38.55% higher than that observed in the untreated group. For the fluorescent dust treatment, the hazard ratio is 1.122, indicating a 12.16% higher hazard. In contrast, the hazard ratio for the rubidium treatment is 0.881, indicating an 11.92% lower hazard. Despite the aforementioned hazard ratios, the *P*‐values associated with the treatment coefficients are all greater than 0.05 (0.468, 0.801, 0.784), indicating that none of these coefficients are statistically significant. Therefore, there is no strong evidence that any of the treatments have a different effect on survival compared to the untreated group.

**Table 3:** Coefficient (coef), exponentiated coefficients (exp(coef)), standard errors (se(coef)), *z*‐values, and *P*‐values from the Cox proportional hazards model assessing the effect of different treatments on females treated survival over time

	coef	exp(coef)	se(coef)	*z*	*P*‐value
Fluorescein	0.326	1.386	0.449	0.726	0.468
Fluorescent dust	0.115	1.122	0.454	0.253	0.801
Rubidium	−0.127	0.881	0.464	−0.274	0.784

**Fig. 1:**
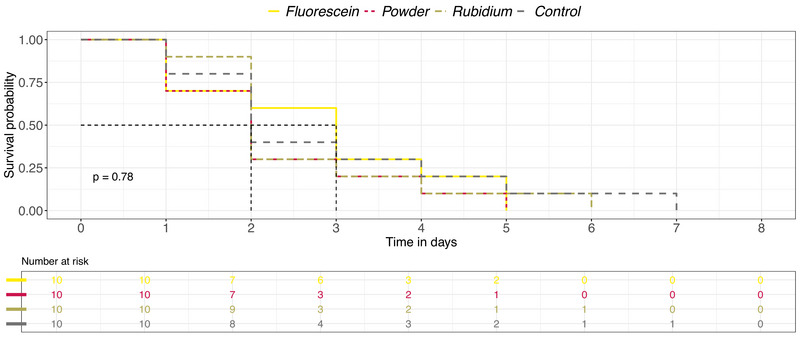
Kaplan–Meier survival curves showing the probability of survival over time (in days) for treated (fluorescein, rubidium, and dust) and untreated (control) hoverflies with different line types distinguishing between treatments. A risk table below the plot summarizes the number of individuals at risk for each treatment at specific time points.

Additionally, the Kaplan–Meier plots and corresponding risk tables, shown in Fig. 1, illustrate the survival curves observed in the different treatments. The absence of significant differences in survival over time between treatments was confirmed by the log‐rank test (*P* = 0.78).

### Marking persistence

**Fig. 2:**
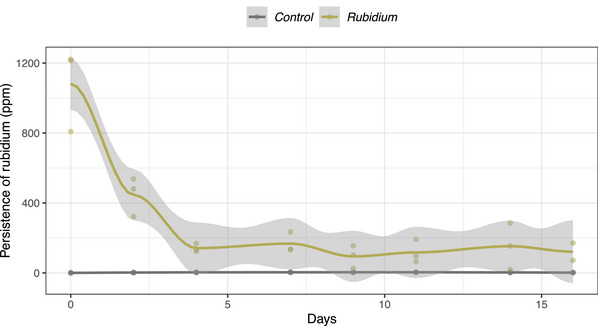
Persistence of rubidium (ppm) in the experimental group (Rubidium) compared to the control group, measured over 16 d. The points represent the observed values, while the shaded area indicates the 95% confidence interval.

Following the marking procedure and over an observation period of 16 d, the natural Rb content in untreated females exhibited a range of variation from a minimum of 0.39 ppm to a maximum of 5.24 ppm, with a mean of 2.75 ppm (± 1.31 ppm SD, *n* = 24). In RbCl‐treated females (*n* = 23), the initial rubidium content ranged from a minimum of 807.79 ppm to a maximum of 1220.90 ppm on the first day after treatment. It then decreased to values between 321.98 ppm and 536.95 ppm after 2 d and finally stabilized at levels between 19.19 ppm and 285.25 ppm, with an average of 133.18 ppm for the rest of the experimental period. The persistence curves of Rb in RbCl‐treated and untreated females are shown in Fig. 2.

**Fig. 3:**
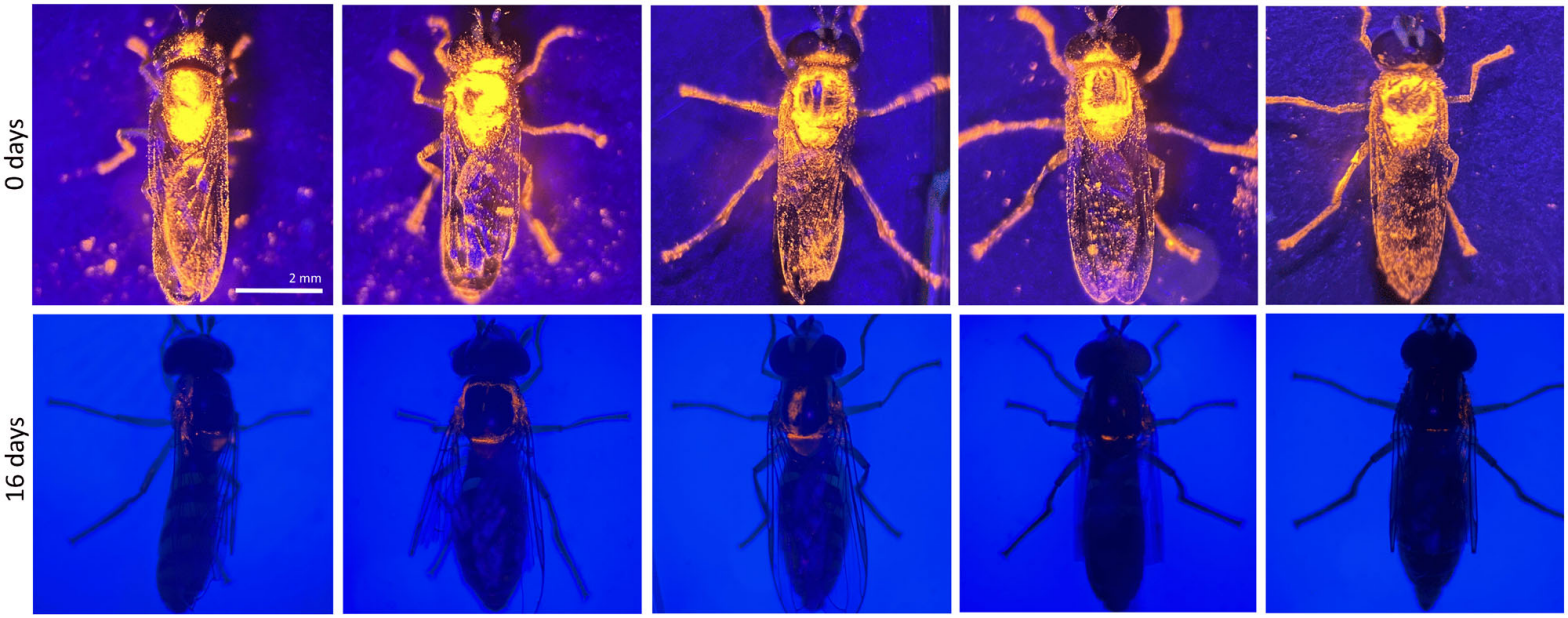
Examples of fluorescent dust‐treated females of *Sphaerophoria rueppellii* at the beginning and at the end of the trial.

**Fig. 4:**
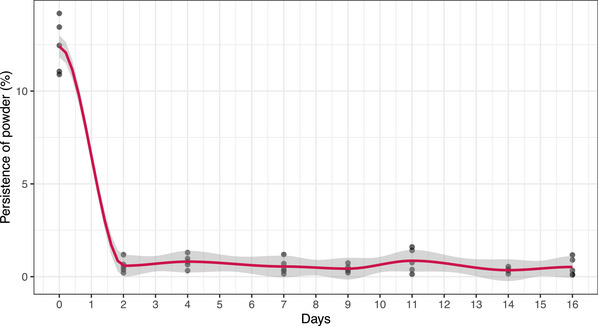
Persistence of the fluorescent dust (%) over 16 d. The points represent the observed values, while the smooth line indicates the 95% confidence interval.

Despite a reduction in the body area covered by the dust from the time of marking until only the second day after marking, fluorescent dust was detected in every hoverfly for the entire duration of the experiment (16 d after marking). From the second to the sixteenth day after marking, between 2.8% and 6.9% of the initial body area covered by dust remained visible. By the end of the observation period, traces of dust were observed in areas of the body that are less accessible during cleaning, typically at the suture between the mesonotum and scutellum and at the attachment site of the wings (Fig. 3). The trend in dust persistence, expressed as a percentage of the area showing fluorescence in the images of marked females, is shown in Fig. 4.

**Fig. 5:**
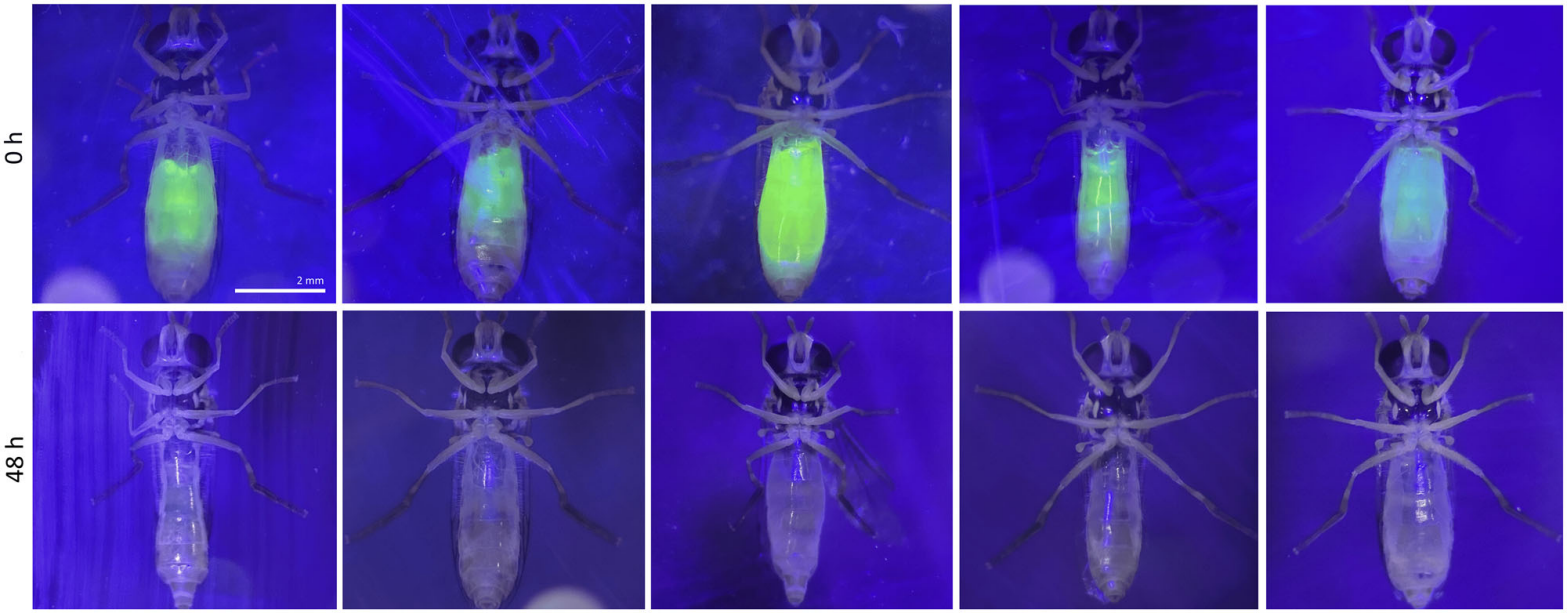
Examples of fluorescein‐treated females of *S. rueppellii* at the beginning and 48 h after marking.

In the case of fluorescein, 100% of the females exhibited fluorescence at the end of the marking period (time 0). After 24 h, 50% of the females could still be considered marked. However, after only 48 h, none of the females showed any fluorescence (Fig. 5).

## Discussion

The present study sought to assess additional marking methods for elucidating the movements of adult predatory hoverflies. Fluorescein dye, fluorescent dusts, and the trace element rubidium were selected for this investigation because they have been extensively employed for tracking other insects with favorable outcomes. Regarding the effects of the marking methods tested here on insect longevity and reproduction, our results are consistent with those of similar studies. In general, larvae of several Lepidoptera species reared with RbCl levels up to 5000 ppm appear to show no adverse effects on the longevity and fecundity of the emerged adults (Polavarapu *et al.*, 1992), and concentrations up to 18 000 ppm RbCl in foliar sprays had no effect on mortality or development of the black‐faced leafhopper *Graminella nigrifrons* (Forbes, 1885) (Hemiptera: Cicadellidae) (Alverson *et al.*, 1980). With regard to Diptera, adult of tiger mosquito, *Aedes albopictus* (Skuse 1894) (Diptera: Culicidae), fed on blood with RbCl concentrations ranging from 0.015 mol/L to 0.5 mol/L showed no differences in egg production and survival compared to the control group, at least during the three weeks following the labeled blood meal (Maciel‐de‐Freitas *et al.*, 2004). Similarly, the mean adult longevity of the Mediterranean fruit fly, *Ceratitis capitata* (Wiedemann, 1824) (Diptera: Tephritidae), did not differ significantly when fed a diet with no added Rb, 1000, 5000, or 10 000 *µ*g Rb/g throughout life (Van Steenwyk *et al.*, 1992). Although in our experiment the median egg count in the rubidium treatment was substantially higher (63 eggs vs. 2 eggs in other treatments), the lack of statistical significance can be attributed to high within‐group variability and a small effect size (*ϵ* ^2^ = 0.0488), suggesting that although there were observable differences, they were modest and not large enough to reach statistical significance. A *post hoc* power analysis confirmed that the study had sufficient power (0.96), suggesting that the sample size was adequate to detect meaningful differences. Future studies with larger sample sizes will be useful to confirm these findings and provide more robust conclusions regarding the effects of treatments on egg production. Moreover, the relatively low concentration of RbCl adopted demonstrated satisfactory persistence, as rubidium levels in treated females remained consistently higher than in untreated ones throughout their entire lifespan. Stimmann (1974) established a baseline for detecting rubidium‐marked individuals in field studies as any concentration above the mean + 3 SD of unmarked insects. According to our results, this threshold level would be 6.69 ppm. All RbCl‐unmarked *S. rueppellii* would be considered unmarked (the maximum Rb level found in RbCl‐unmarked females was 5.24 ppm) and all RbCl‐marked hoverflies would be considered as marked (the minimum Rb level found in RbCl‐marked females was 19.19 ppm), confirming that a concentration of 0.01 mol/L RbCl is sufficient to successfully mark *S. rueppellii*.

Fluorescent dusts are widely used to mark insects, and their application has been reported to range from having no adverse effects to being detrimental to the survival of the specimens. Focusing only on Diptera, fluorescent dusts were found to be indifferent to the survival of adult *Drosophila pseudoobscura* (Frolova, 1929) (Crumpacker, 1974) (Diptera: Drosophilidae), adult of *Anopheles gambiae* (Giles, 1902) (Verhulst *et al.*, 2013) (Diptera: Culicidae) and adult of spotted wing Drosophila, *Drosophila suzukii* (Matsumura, 1931) (Clymans *et al.*, 2020) (Diptera: Drosophilidae). Conversely, several factors can cause side effects when using dusts. These include the chemical nature of the dust (Rojas‐Araya *et al.*, 2020), the amount of dust applied (Dominiak *et al.*, 2010; Makumbe *et al.*, 2017) and the application technique (Dickens & Brant, 2014). The application method employed in this study is more time‐consuming and less standardizable than the most commonly used method, which involves placing the insects in a container with a specific amount of dust and shaking the container. However, our method allows only a small amount of dust to be applied, sufficient to effectively mark the insect without an excess that could lead to unwanted/negative side effects. Regarding the persistence of dust over time, the rapid decrease in dust coverage can be attributed to the cleaning behavior of hoverflies, which use their legs and tarsi to remove dust from their bodies. However, although small traces of dust remained visible after just 2 d, the marking dust was clearly discernible throughout the entire observation period, providing satisfactory persistence. Fluorescein is a less widely used insect marker, but all previous work is consistent with our results. Adult of the southern house mosquito, *Culex quinquefasciatus* (Say, 1823) (Diptera: Culicidae), reared on a solution of water and sugar with 0.01% fluorescein did not differ significantly from the control group in terms of survival time and number of eggs laid (Sarkar *et al.*, 2017). Similar tests carried on screw‐worm, *Cochliomyia hominivorax* (Coquerel, 1858) (Diptera: Calliphoridae), showed little effect on adult longevity, with an average 10‐d mortality of adults fed a diet of corn syrup‐water mixed with 0.01%, 0.1%, and 1.0% of fluorescein being 81%, 71%, and 74%, respectively, compared with an average mortality of 72% for adults fed with control diet (Coppedge *et al.*, 1979). On the contrary, the short persistence obtained with fluorescein in our experiment contrasts with previous studies. Adult *C. hominivorax* fed a diet containing 0.01% fluorescein for only 6 h showed fluorescence in the 26% of cases after 10 d (Coppedge *et al.*, 1979). However, the detection of fluorescein in this study was assessed by crushing the flies, especially with increasing time after marking. Our intention was to use fluorescein as a nondestructive marker that would allow detection in live insects in the field. However, it is possible that differences in physiology or metabolism between *C. hominivorax* and *S. rueppellii*, such as different timing of excretion or feeding activity, could result in faster clearance of fluorescein from the body in the hoverfly.

It should be noted that in our results, the mean number of eggs laid per female and the mean female life span are noticeably lower than the reproductive potential and life expectancy of *S. rueppellii* reported in the literature. In Orengo‐Green *et al.* (2022), the maximum number of eggs laid over the entire lifespan (an average of 294.67 eggs per female) was observed under a 14 L: 10 D photoperiod at 25 ± 1 °C, and this number decreased drastically under a different photoperiod and/or at a temperature of 20 ± 1 °C. The maximum female lifespan (29.6 d on average) was observed with a photoperiod of 12 L: 10 D at 20 ± 1 °C and decreased with rising temperature. Our tests were conducted under a 16 L: 8 D photoperiod at 26 ± 1 °C, which may explain our results.

Understanding the effects of markers on mating behavior is more complex because mating was observed in only 44%, 45%, and 68% of experimental replicates (for dust, rubidium and fluorescein, respectively). Behaviors associated with mate location in insects could be various and challenging to study. They include station taking (a male watches for females from a vantage point such as a leaf or tree trunk, or while hovering over or under a visible marker) (Downes, 1969); territoriality (a male defends the area around his station from other males); hilltopping (both sexes orient to some high topographic feature, where the males take stations) (Alcock, 1987); lek formation (several males compete for females by defending small territories within an arena that offers the females no resources other than the males themselves) (Alcock, 1981); and other mate‐seeking behaviors, including flower patrolling (males wait on the foliage of flowering plants or near oviposition sites) and hovering (males fly near flowers and other resources used by females). All of these behaviors have been reported for hoverflies (Chapman, 1954; Collett & Land, 1975a; Collett & Land, 1975b; Maier, 1978; Maier & Waldbauer, 1979; Fitzpatrick & Wellington, 1983; Gilbert, 1984; Waldbauer, 1990). In our mating behavior tests, we have opted for extreme simplification and standardization in order to investigate the choice of a male over a marked or unmarked female, or the mate‐seeking ability of a marked female over an unmarked one. Therefore, all of the above‐mentioned mating behaviors that were not included in these tests may have played a key role, and further and more structured investigations are needed. Also, maturation of the testes and accessory glands in syrphid males is required to be able to fertilize (and maybe consequently mate with) the female (Gilbert & Falk, 2015), but this cannot explain the low number of copulae observed, as in Orengo‐Green *et al.* (2022) most males held at 25 °C copulated at 2 d of age and females laid fertile eggs the next day, whereas in our tests both males and females were at least 3 d old. It is noteworthy that the mating choices in the fluorescent dust treatment tests differed significantly from those in the fluorescein and rubidium tests. It is possible that the dust affected the mobility and activity of the females, resulting in a reduction in their ability to seek males or their attractiveness to males. Indeed, dust marking has been shown to affect insect behavior in several cases. In Moffitt & Albano (1972), two types of dust affected the ability of released marked males of codling moth, *Cydia pomonella* (Linnaeus, 1758) (Lepidoptera: Tortricidae), to find females in an orchard and greenhouse. Dominiak *et al.* (2010) reported negative effects of a dust pigment on the flight ability of the Queensland fruit fly, *Bactrocera tryoni* (Froggatt, 1897) (Diptera: Tephritidae). Rojas‐Araya *et al.* (2020) found that dust affected the ability of marked females of the yellow fever mosquito, *Aedes aegypti* (Linnaeus, 1762) (Diptera: Culicidae), to enter baited traps, possibly by interfering with sensory organs related to olfaction. It is also possible that marked females are less attractive to males due to the visibility of the orange dust, which makes the females appear anomalous compared to the average. Photoreceptors for UV light have long been known to exist in hoverflies (Bishop, 1974). *Eristalis tenax* (Linnaeus, 1758) has shown a preference for yellow‐colored disks based on their UV light absorption or reflectance (An *et al.*, 2018). There is also evidence suggesting that *Eristalis arbustorum* (Linnaeus, 1758) may avoid flowers treated with neonicotinoids that reflect specific wavelengths of light (Clem *et al.*, 2020). Therefore, it is possible that the fluorescent dust's ability to reflect UV light could have influenced the choices of males ultimately; it must be considered that dust could interfere with the mate‐seeking activity of hoverflies in the field, with a consequent reduction in fitness.

In conclusion, we have shown that rubidium, fluorescein and fluorescent dusts can be effective markers for *S. rueppellii* as they meet one of the main requirements for a good marker, that is, they should not disrupt the insect normal biological functions. Specifically, we found no evidence that any of the techniques used in this study, at the concentrations and application methods tested, affected the fecundity and longevity of *S. rueppellii*. However, while fluorescein and rubidium did not influence the mating behavior of hoverflies, fluorescent dust‐treated females mated significantly less, suggesting an interference with mating behavior that warrants further investigation. Finally, differences in marking persistence and application methods suggest that each technique may be suitable for different purposes. Rubidium showed good long‐term persistence, but its detection is time‐consuming, requires specialized equipment and trained personnel, and involves a destructive process that requires killing the insect. Fluorescein and fluorescent dust are cost‐effective and easy‐to‐use options; however, fluorescein showed very short persistence, making it suitable only for short‐term MRR programs. While this study provides valuable insights into the effectiveness of three marking methods for hoverflies, one limitation is the relatively small sample sizes in several experiments. This constraint may reduce the statistical power to detect small or subtle effects, thereby necessitating cautious interpretation of the findings. Future research with larger sample sizes and broader experimental conditions would be beneficial to validate these findings and explore potential variability in marking effectiveness.

## Disclosure

The authors declare that the research was conducted in the absence of any commercial or financial relationships that could be construed as a potential conflict of interest.
